# Prevalence of Human Parvovirus B19, Bocavirus, and PARV4 in Blood Samples from the General Population of China and Lack of a Correlation between Parvovirus and Hepatitis B Co-Infection

**DOI:** 10.1371/journal.pone.0064391

**Published:** 2013-05-30

**Authors:** Rui Tong, Liping Shen, Wenjiao Yin, Weimin Zhou, Jian Lu, Meiqin Zheng, Shengli Bi, Yongliang Lou, Wenjie Tan

**Affiliations:** 1 Institute of Medical Virology, Wenzhou Medical College, Zhejiang, China; 2 Key Laboratory of Medical Virology, Ministry of Health, National Institute for Viral Disease Control and Prevention, China CDC, Beijing, China; 3 Eye Hospital, Wenzhou Medical College, Zhejiang, China; University of Illinois at Chicago, United States of America

## Abstract

Few comprehensive studies have investigated viraemia caused by human parvoviruses (HPAVs) in China. A total of 1626 of blood samples were collected from non-HBV and HBV infected Chinese subjects (adults, N = 1279; children, N = 347) from south-western and south-eastern China. DNA from three HPAVs was detected in blood samples using PCR-based assays. The epidemiological profiles and association with HBV co-infection were also analysed. Of the 1626 blood samples tested, 138 (8.49%) were found to exhibit HPAV viraemia, including 3.51% with B19, 3.75% with HBoV and 2.52% with PARV4. The presence of B19 DNA in both child and adult, as well as that of PARV4 DNA in adult,from the south-western region was significantly higher than that from the south-eastern region (*P* = 0.006 for B19 in children; *P* = 0.026 for B19 in adults; and *P* = 0.014 for PARV4 in adult).However, the frequency of HBoV DNA in adults from the south-western region was significantly lower than that observed in adults from the south-eastern region (*P = *0.001). Furthermore, HBoV was more prevalence in male (4.9%) than in female (1.4%) individuals. In addition, no significant correlation between HBV and HPAV co-infection was found using serum samples from Chinese adults. In conclusions,the molecular prevalence of three HPAVs in blood samples exhibited variation among different populations depending on area, age and gender; No association between HPAV and HBV infection in adults was found. Our data provide a basis for improving blood safety and preventing HPAV infection in China.

## Introduction

Human parvoviruses (HPAVs) are small, non-enveloped, single-stranded DNA viruses. In recent years, the number of identified HPAVs has increased rapidly [1]. In addition to B19, which was the first HPAV known to cause erythematous infectiosum and a variety of other disease manifestations [2], the HPAV family now also includes human bocavirus (HBoV) and parvovirus 4 (PARV4), both of which were first reported in clinical samples in 2005 [3], [4]. Several studies have shown that HBoV was detected in human respiratory secretions and faeces and might associate with acute respiratory tract symptoms and gastrointestinal disease [5]–[7]. However, HBoV has also been frequently detected in asymptomatic patients and in patients co-infected with other pathogens [7]. PARV4 has been detected in blood, pooled plasma, and diverse tissue samples of HIV/AIDS patients who are also known injectable drug users [8], [9]. However, the clinical significance of co-occurrence of PARV4 DNA in hepatitis B virus (HBV)-infected patients remains controversial [8]–[12].

Previous studies have suggested that B19, HBoV and PARV4 are the pathogenic members for human among parvovirus and likely to circulate globally [7], [11], [13]–[19]. Both HBoV and PARV4 was recently identified novel HPAVs. Little is known about the mode and frequency of transmission and their full pathogenicity of HBoV and PARV4 infection. Up-to-date, none reported the epidemiological profile of HBoV and PARV4 infection among general populations in China. In addition, few studies of HBoV DNA in blood samples are available [7], [16], [19], [20].

In addition, the clinical significance of HPAV occurrence in patients co-infected with hepatitis remains controversial [14]–[16]. Co-infection of parvovirus B19 and PARV4 with HBV or hepatitis C (HCV) has been observed in patients with acute and chronic hepatitis [17], [18]. In the southern region of China, HBV infection is endemic, with an HBsAg seroprevalence of approximately 7–9% [21], [22]; however, the seroprevalence of HPAV among the HBV infection individuals in this area is unknown.

The goal of the present study was to determine the prevalence and epidemiological profiles of various HPAVs among populations in southern China. We screened blood samples using PCR-based assays to investigate the association of three HPAVs (B19, HBoV and PARV4) in healthy subjects and HBV-infected individuals; we also evaluated symptomatic HBV patients *versus* asymptomatic HBV carriers in southern China.

## Methods

### Ethical Statement

All human sera were de-identified before use in the study, and all laboratories testing of samples were approved by the Ethical Review Committee of the National Institute for Viral Disease Control and Prevention (CNVCDC) and the Wenzhou Medical College (WMC). All work involving human blood samples was approved by the Ethical Review Committee of the CNVCDC and the WMC. The blood samples were collected as part of routine surveillance activities undertaken by CNVCDC and WMC, and so consent was waived by the Ethical Review Committee.

### Study Groups

**Figure 1:**
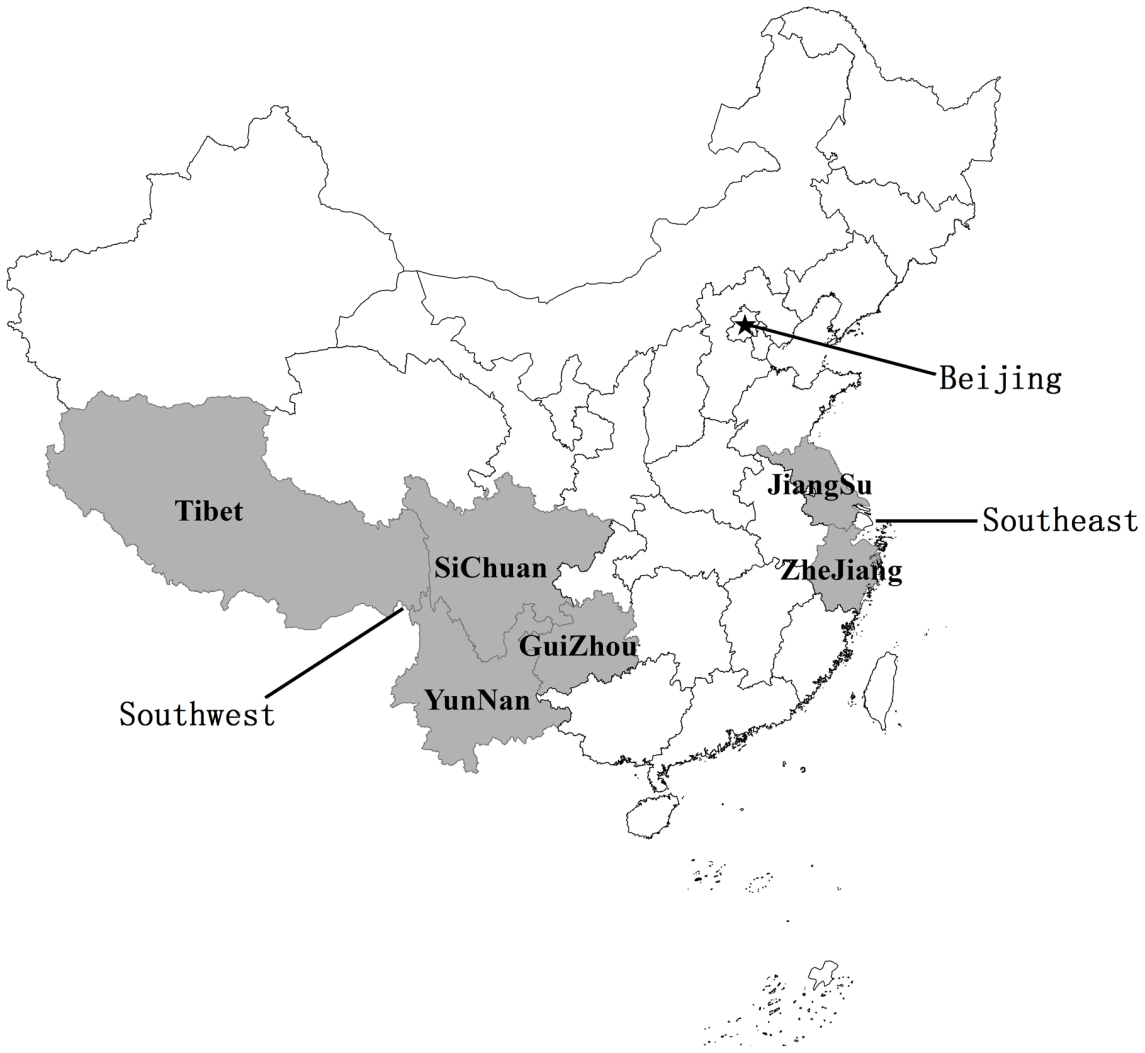
Location of samples collected for screening of three human parvoviruses (HPAVs) in China.

This study involved a total of 1626 blood samples from the southern region of China (Table 1). Samples were divided into groups as the southwest area(including several provinces such as Chongqing, Guizhou, Yunnan, Sichuan and Xizang) representing multi-ethnic and less-developed zone, and the southeast (Zhejiang and Jiangsu province) areas representing the costal and developed zone in China(Figure 1).The sample size and demographic characteristics of all participants were matched between southwest and southeast area. All samples were collected from health examination population and retrieved randomly from the Sample Bank of the CNVCDC and the WMC from 2005 to 2012. Serum specimens were tested for the presence of HBV, HCV and HIV-1 viral genomes using commercial real-time PCR kits. None were positive for HIV-1 or HCV RNA. HBsAg and HBeAg were assayed using commercial ELISA kits (Kehua Corporation, Shanghai). In this study, we used 347 serum samples from children and 1279 from adults. The cohort included the following: samples defined as healthy controls were serum-negative for HBV DNA, HCV and HIV-1 RNA; samples that were serum-positive for HBV DNA and HBV surface antigen (HBsAg) but negative for HBV e antigen (HBeAg) were termed HBV carriers; samples that were serum-positive for both HBsAg and HBeAg were termed chronic HBV infected patients. All serum samples from the Xizang area were Tibetan in origin; samples from the Sichuan areas were Han in origin.

**Table 1:** Demographic characteristics of all participants (N = 1626).

Group	Characteristics	Number	Percentage (%)
**Gender**	Male	966	59.4
	Female	660	40.6
**Age (year)**	Children (1–14)	347	21.3
	Adults (18–81)	1279	78.7
**Area**	Southwest	741	45.6
	Children	175	
	Adults	566	
	Southeast	885	54.4
	Children	172	
	Adults	713	
**Cohort in Adults**	HBV DNA(−)	563	
	HBV DNA(+)	716	
	HBsAg^+^ HBeAg^−^	400	
	HBsAg^+^ HBeAg^+^	316	
**HPAV DNA(+)**	Any	138	8.49
	B19	57	3.51
	HBoV	61	3.75
	PARV4	41	2.52

### Detection of B19, HBoV and PARV4/5

Nucleic acid was extracted from 200 µL samples of blood using QIAamp MinElute Virus Spin Kit (Qiagen, Mississauga, Ontario, Canada) according to the manufacturer’s instructions. Aliquots (10 µL) of the nucleic acids were subjected to a nested PCR assay for three HPAVs (B19, HBoV and PARV4), as described previously [11], [13]. The primers used in this study are listed (Table S1). PCR amplicons for DNA sequencing were gel purified using the PCR Clean-Up System (Promega, Madison, Wisconsin) according to the manufacturer’s instructions. DNA sequencing was performed with specific primers using the ABI PRISM BigDye Terminator Cycle Sequencing Reaction kit (version 3.1) on an ABI PRISM 3130 DNA sequencer (Applied Biosystems, Foster City, California) following the manufacturer’s instructions.

### Statistical Analysis

Eligibility and classification of samples were determined from the original medical record in the database. Univariate associations between age and the frequency distribution of viral pathogens were analysed by logistic regression. Univariate associations between the distributions of virus and co-detections and the clinical picture of illness were assessed by multinomial logistic regression analysis. Statistical significance was assessed by one-way ANOVA followed by Tukey’s test. The statistical analyses were performed using SAS (version 9.2; SAS Institute, Cary, NC, USA). *P* values <0.05 were considered to indicate statistical significance.

## Results

### Characteristics of Study Participants

The general demographic information of the subjects is listed in Table 1. Groups were formed based on gender, age, area, population, and HBV infection status among adults. Overall, a total of 1626 blood samples were screened for B19, HBoV and PARV4 in this study. A total of 966 (59.4%) samples were from male subjects and 347 (21.3%) were from children (all child samples tested negative for HBV). A total of 741 samples were collected from the population in the south-western region of China, including samples from 175 children and 566 adults; 885 samples were collected from the population in the south-eastern region of china, including samples from 172 children and 713 adults. A total of 125 samples were collected from the Tibetan population in Xizang and 528 samples were from the Han population in the Sichuan province. Cohorts were formed among healthy adult subjects (HBV negative, N = 563) and HBV-infected subjects (HBV positive, N = 716); the group was further divided into HBV carriers (N = 400, HBsAg^+^ HBeAg^−^) and chronic HBV patients (N = 316, HBsAg^+^ HBeAg^+^).

### Distribution of HPAV Infection by Age, Gender, Area, and Population

**Figure 2:**
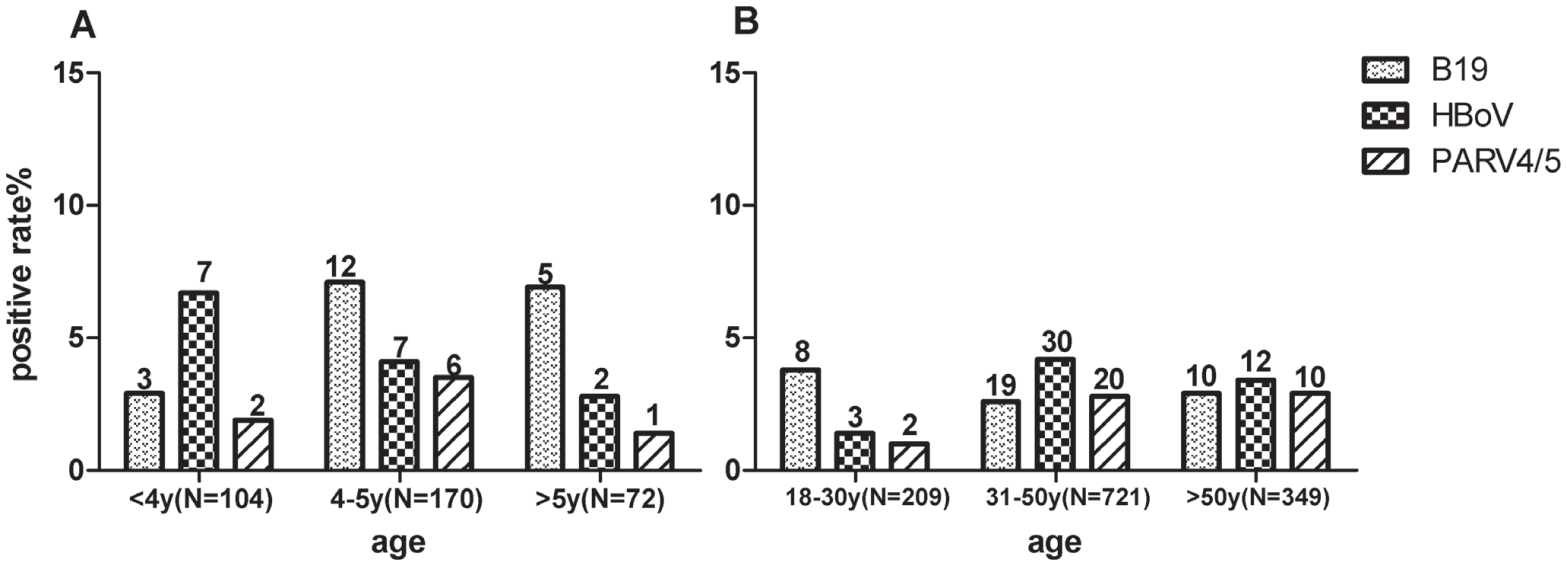
Frequencies of B19, HBoV and PARV4 occurrence in blood samples from different age groups.

All blood samples were screened using nested PCR for the DNA of three HPAVs: B19, HBoV and PARV4. Of 1626 blood samples tested, 138 (8.49%) exhibited HPAV infection, including 57 (3.51%) with B19, 61 (3.75%) with HBoV, and 41 (2.52%) with PARV4 (Table 1). We further analysed the distribution of HPAV infection by age, gender, area, and population. As shown in Table 2, we detected 20 (5.76%) with B19, 16 (4.61%) with HBoV, and nine (2.6%) with PARV4 in the child group (N = 347). There was no significant difference in the prevalence of the three HPAVs between male and female subjects. In addition, no significant difference in the prevalence of HBoV and PARV4 infection between subjects from the south-western and south-eastern regions was observed. However, the B19 positivity rate (9.1%) among children from the south-western region was significantly higher than that (2.3%) in children from the south-eastern region (*P* value = 0.006). The prevalences of the three HPAVs in three age groups (<4 years, 4–5 years, and >5 years) were also determined (Figure 2). B19 and PARV4 infections increased among the 4–5-year-old group compared to those under the age of 4 years. However, HBoV positivity peaked among young children and then decreased with advancing age (6.73% for the <4 years group, 4.12% for the 4–5 years group, and 2.78% for the >5 years group).

**Table 2:** Prevalences of B19, HBoV and PARV4 in different age, gender, and regional groups.

Group	B19	HBoV	PARV4
**Children (N = 347)**	**20 (5.76%)**	**16 (4.61%)**	**9 (2.6%)**
**Gender**			
Male (N = 193)	14 (7.3%)	10 (5.2%)	7 (3.6%)
Female (N = 154)	6 (3.9%)	6(3.9%)	2 (1.3%)
*P* value	0.182	0.571	0.31
**Region**			
Southwest (N = 175)	16 (9.1%)	8 (4.6%)	7 (4.0%)
Southeast (N = 172)	4 (2.3%)	8 (4.7%)	2 (1.2%)
*P* value	**0.006**	0.972	0.185
**Adults (N = 1279)**	**37 (2.89%)**	**45(3.52%)**	**36 (2.50%)**
**Gender**			
Male (N = 773)	23(3.0%)	38 (4.9%)	16 (2.1%)
Female (N = 506)	14 (2.8%)	7 (1.4%)	16 (3.2%)
*P* Value	0.828	**0.001**	0.221
**Region**			
Southwest (N = 566)	23 (4.1%)	9 (1.6%)	21 (3.7%)
Southeast (N = 713)	14 (2.0%)	36(5.0%)	11 (1.5%)
*P* value	**0.026**	**0.001**	**0.014**

In the adult sample group (N = 1279), we detected 37 (2.89%) with B19 infection, 45 (3.52%) with HBoV infection, and 32 (2.50%) with PARV4 infection. No significant difference was observed in B19 and PARV4 infection between male (N = 773) and female (N = 556) subjects. However, the HBoV positivity rate (4.9%) in males was significantly higher than that (1.4%) in females. In addition, the prevalence of the three HPAVs exhibited significant differences between subjects from the south-western and south-eastern regions of China. Both B19 and PARV4 were more prevalent in south-western region samples (B19, 4.1% *vs.* 2.0%, *P* = 0.026; PARV4, 3.7% *vs.* 1.5%, *P* = 0.014). In contrast, HBoV was more prevalent in south-western region subjects(5.0% *vs.* 1.6%, *P* = 0.001). The prevalence of three HPAVs among three adult age groups (18–30 years, 31–50 years, and >50 years) was also analysed (Figure 2). Both HBoV and PARV4 exhibited significantly lower infection rates (<1.43%) than B19 (3.82%) among the 18–30-year-old group.

Two populations (Han and Tibetan) from adjacent provinces (Sichuan and Xizang) in the south-western region were evaluated to assess the HPAV infection rates among ethnicities (Table 3). Among 528 subjects from the Sichuan Han population, we detected 17 (3.2%) with B19, nine (1.7%) with HBoV, and seven (1.3%) with PARV4. Among 125 subjects from the Xizang Tibetan population, we detected six (4.8%) with B19, none with HBoV, and 12 (9.6%) with PARV4. The prevalence of PARV4 in the Xizang Tibetan population was significantly higher than that in the Sichuan Han population.

**Table 3:** Prevalences of B19, HBoV and PARV4 in Han and Tibetan populations in the south-western region.

	B19	HBoV	PARV4
Han (Sichuan = 528)	17 (3.2%)	9 (1.7%)	7 (1.3%)
Tibetan (Xizang = 125)	6 (4.8%)	0	12 (9.6%)
*P* value	0.554	0.297	**0.001**

A total of 16 cases with HPAV co-infection were identified (Table S2). Half of these were children, five of which exhibited co-infection with all three HPAVs (B19, HBoV and PARV4).

### Comparison between Healthy and HBV Infection Adults

To explore the correlation between HPAV and HBV co-infection, we formed cohorts of healthy (HBV-negative) and HBV-infected adults; the HBV-infected group was further divided into HBV carriers (HBsAg^+^ HBeAg^−^) and chronic HBV patients (HBsAg^+^ HBeAg^+^) (Table 4). The B19 and PARV4 detection frequencies were similar in both healthy and HBV-infected adults. However, the HBoV positivity rate (6.6%) in healthy adults was higher than that (1.1%) in HBV-infected adults (Table 4). In addition, no significant difference in the prevalence of HPAVs between HBV carriers and chronic HBV patients was observed.

**Table 4:** Prevalences of B19, HBoV and PARV4 among healthy adults (HBV DNA negative), HBV carriers (HBsAg^+^ HBeAg^−^) and chronic HBV patients (HBsAg^+^ HBeAg^+^).

Group	B19	HBoV	PARV4
HBV DNA(−) (N = 563)	18 (3.2%)	37(6.6%)	16 (2.8%)
HBV DNA(+) (N = 716)	19 (2.7%)	8 (1.1%)	16 (2.2%)
* P* value	0.565	**0.001**	0.490
HBsAg^+^ HBeAg^−^ (N = 400)	10 (2.5%)	3 (0.75%)	8 (2.0%)
HBsAg^+^ HBeAg^+^ (N = 316)	9 (2.9%)	5 (0.41%)	8 (3.3%)
* P* value	0.774	0.488	0.633

## Discussion

The present study is the first comprehensive investigation of the epidemiological profiles of HPAVs among the general population in China using blood samples and PCR-based assays. The overall prevalence of HPAV viraemia in the general Chinese population was 3.51% B19, 3.75% HBoV and 2.52% PARV4. Groups were evaluated based on gender, age, and regional differences. We also compared the frequency of three HPAVs between HBV-infected subjects and healthy controls. We found several aspects of HPAV infection that had not been reported previously, and we increased the knowledge of the epidemiological profiles of HPAV infection in China.

As reported previously, transmission of parvovirus B19 occurs most commonly by personal contact or via blood, sexual intercourse, or a maternal-neonatal relationship, and it is usually associated with an expanding range of clinical disorders [14], [16]. However, it has also been shown that B19 DNA can persist in healthy or immunocompetent individuals for long periods [11], [14], [16]. Few screening studies have evaluated plasma pools, blood products, or clinical specimens with the goal of improving blood product safety and/or determining clinical significance [23], [24]. The overall frequency (3.51%) of B19 DNA in blood samples in the general population of southern China was first reported in this study. B19 was also co-detected in the sera of HBV-infected patients. A significant correlation exists between HBV/B19 co-infection using serum samples from Vietnamese and Nigerian subjects [17], [18]. However, the data in our study did not support the conclusion that B19 infection increased the frequency of HBV co-infection.

A variety of signs and symptoms have been described in patients with HBoV infection [5], [7], [15], [16]. Many of these potential manifestations have not been systematically explored, and they have been questioned due to the high HBoV co-infection rates in symptomatic subjects and high HBoV detection rates in asymptomatic subjects [5], [7], [15], [16]. However, evidence is mounting to show that HBoV1 is an important cause of lower respiratory tract illness, while HBoV2-4 is enteric viruses [7], [15], [16]. HBoV DNA was generally detected in respiratory or faecal samples but only rarely in blood or plasma pools [15], [16]. HBoV DNA was detected in blood samples in 3.75% of general asymptomatic subjects in our study. Interestingly,we found that the HBoV DNA was found more frequently (4.9%) in the blood of male subjects than in that of female subjects (1.4%) among adult population. Using age association, we found that HBoV viraemia peaked in young children (<4 years) and decreased with advancing age in general asymptomatic children. This trend is similar to that in the frequency of acute respiratory or enteric infections in children [15], [16], [20], [25], [26]. We hypothesised that the frequency of HBoV transmission among children decreased with advancing age and that slight or asymptomatic HBoV infection might occur in a few child individuals. Whether HBoV caused persistent infection is unclear and warrants further investigation, such as by comparing the viral loads and the dynamics among confirmed acute cohort cases. Comparison of HBoV detection rates in healthy and HBV-infected adults suggested no correlation between HBV and HBoV infection.

PARV4 is a DNA virus frequently associated with HIV and HCV infections [10], [12], [16], [27], but its clinical significance remains unknown [16], [27]–[30]. The overall frequency (2.52%) of PARV4 viraemia observed in our study is in fairly good agreement with most previous results (0 to 5% in plasma from healthy blood donors) conducted in other areas of the world [10], [12], [16], [28], [29]. However, there are significant differences between our data and that reported in a recent, similar study in Shanghai [27] by Yu *et al.*, in which the PARV4 was the only parvovirus detected and its infection rate was strikely high as 16–22%, 33% and 41% in healthy controls, HCV infected and HBV infected subjects respectively, comparing to previous reports published [8]–[10], [12], [13], [16], [29], [30]. There are several possible explanations for this difference in frequency between our data and Yu’s study. First, the samples were collected from different populations (general *vs.* HCV-infected subjects included); second, different procedures and conditions for PCR assays were used; third, regional (rural or suburban *vs.* metropolis) and temporal (2005 to 2012 *vs.* 2008 and 2009) differences may have influenced the results. To date, no disease has been determined to be associated with PARV4 infection. Our data also suggest no correlation between HBV and PARV4 infection. An interesting finding of this study is that the prevalence of PARV4 in the Xizang Tibetan population was significantly higher than in the Sichuan Han population; the reason(s) for this are unknown and should be explored further using greater numbers of subjects and assessment of other related background information.

The prevalence of HPAV infection differed between populations from the south-western and south-eastern regions of China. The presence of B19 DNA in blood samples of both children and adults from the south-western area was significantly higher than that observed in samples from south-eastern region subjects (*P* = 0.006 for children; *P* = 0.026 for adults). In addition, PARV4 was more prevalent in adults from the south-western region (3.7% *vs.* 1.5%, *P* = 0.014), while HBoV was more prevalent in adults from the south-western region(5.0% *vs.* 1.6%, *P* = 0.001). The reason for these differences is unclear and warrants future investigation.

Sixteen cases exhibited HPAV co-infection, which suggests that HPAVs share common or similar transmission routes. HPAV co-infection in the general population indicates that HPAVs exhibit minor pathologic effects and that co-infection may not necessarily increase the severity of a clinical disease manifestation.

In summary, our data demonstrate that three HPAVs (B19, newly discovered HBoV, and PARV4) are frequently detected in blood samples and are circulating in both general and HBV-infected populations in southern China. However, the clinical relevance of HPAV occurrence remains unknown. No significant correlation between HBV and HPAV infection in serum samples of Chinese adults was observed. Furthermore, our data suggest that the frequency of HPAV occurrence in blood samples varies according to age, gender, region, and ethnicity. Although the pathologic role of HPAVs remains unclear, the comprehensive epidemiological profiles presented in this study provide a basis for improving blood safety and preventing HPAV infection in China.

## Supporting Information

Table S1 **Primers and thermal profiles of PCR assays for B19, HBoV and PARV4.**
(DOCX)

Table S2 **Co-detection of B19, HBoV and PARV4 DNA in blood samples.**
(DOCX)
