# Supplementary material for: Prevalence of Human Parvovirus B19, Bocavirus, and PARV4 in Blood Samples from the General Population of China and Lack of a Correlation between Parvovirus and Hepatitis B Co-Infection
Source: PLoS One. 2013 May 30;8(5):e64391. doi: 10.1371/journal.pone.0064391 (PMC3667789; doi:10.1371/journal.pone.0064391)
Supplement: Table S1 — Primers and thermal profiles of PCR assays for B19, HBoV and PARV4. (DOCX) [file pone.0064391.s001.docx]

| **Primer** | **Sequence^*^ (5’ – 3’)** | **Target genes** | **Thermal profiles** |
| --- | --- | --- | --- |
| **B19** |  | VP1 | Same profiles for B19, HBoV and PARV4/5：  **1^st^ round:**  94°C for 5 min;  94°C for 30 s,  55°C for 30 s,  72°C for 1 min,  35 cycles;  72°C for 5 min  **2^nd^ round:**  Same as 1^st^ round |
| 1-F | GCTGTTAAGGATGTTACAGA |  |  |
| 1-R | GGATCCGTATAAGGGATTGT |  |  |
| 2-F | CAGGTTACTGACAGCACTAC |  |  |
| 2-R | TGTTGACTGCAGCCCTCTAA |  |  |
| **HBoV** |  | NS |  |
| 1-F | TATGGGTGTGTTAATCATTTGAAYA |  |  |
| 1-R | GTAGATATCGTGRTTRGTKGATAT |  |  |
| 2-F | AACAAAGGATTTGTWTTYAATGAYTG |  |  |
| 2-R | CCCAAGATACACTTTGCWKGTTCCACCC |  |  |
| **PARV4** |  | ORF1 |  |
| 1-F | AAGACTACATACCTACCTGTG |  |  |
| 1-R | TGCCTTTCATATTCAGTTCC |  |  |
| 2-F | GTTGATGGYCCTGTGGTTAG |  |  |
| 2-R | CCTTTCATATTTCAGTTCCTGTTCAC |  |  |

*Y = C/T, R = A/G, K = G/T, W = A/T
