# Supplementary material for: Prevalence of Human Parvovirus B19, Bocavirus, and PARV4 in Blood Samples from the General Population of China and Lack of a Correlation between Parvovirus and Hepatitis B Co-Infection
Source: PLoS One. 2013 May 30;8(5):e64391. doi: 10.1371/journal.pone.0064391 (PMC3667789; doi:10.1371/journal.pone.0064391)
Supplement: Table S2 — Co-detection of B19, HBoV and PARV4 DNA in blood samples. (DOCX) [file pone.0064391.s002.docx]

| **Sample Number** | **Region** | **Years/Gender** | | **HBV Ag** | **B19** | **HBoV** | **PARV4** |
| --- | --- | --- | --- | --- | --- | --- | --- |
| **90** | **ZJ** | **3y** | **male** | **-** | **-** | **+** | **+** |
| **43** | **SC** | **4y** | **male** | **-** | **+** | **+** | **+** |
| **109** | **SC** | **4y** | **female** | **-** | **+** | **+** | **+** |
| **131** | **SC** | **4y** | **female** | **-** | **+** | **+** | **-** |
| **17** | **SC** | **5y** | **male** | **-** | **+** | **-** | **+** |
| **79** | **SC** | **5y** | **male** | **-** | **+** | **+** | **-** |
| **110** | **SC** | **5y** | **male** | **-** | **+** | **+** | **+** |
| **128** | **SC** | **5y** | **male** | **-** | **+** | **+** | **+** |
| **1** | **SC** | **33y** | **male** | **-** | **+** | **-** | **+** |
| **61** | **SC** | **41y** | **male** | **-** | **-** | **+** | **+** |
| **161** | **SC** | **41y** | **female** | **-** | **+** | **+** | **+** |
| **358** | **ZJ** | **45y** | **male** | **-** | **-** | **+** | **+** |
| **56** | **ZJ** | **47y** | **male** | **-** | **-** | **+** | **+** |
| **65** | **ZJ** | **49y** | **male** | **-** | **+** | **+** | **-** |
| **421** | **XZ** | **49y** | **female** | **HBsAg^+^ HBeAg^+^** | **+** | **-** | **+** |
| **16** | **SC** | **67y** | **female** | **HBsAg^+^** | **+** | **+** | **-** |
